# Enhanced case management can be delivered for patients with EVD in Africa: Experience from a UK military Ebola treatment centre in Sierra Leone

**DOI:** 10.1016/j.jinf.2017.12.006

**Published:** 2018-04

**Authors:** S.J. Dickson, K.A. Clay, M. Adam, C. Ardley, M.S. Bailey, D.S. Burns, A.T. Cox, D.G. Craig, M. Espina, I. Ewington, G. Fitchett, J. Grindrod, D.E. Hinsley, S. Horne, E. Hutley, A.M. Johnston, R.L.C. Kao, L.E. Lamb, S. Lewis, D. Marion, A.J. Moore, T.C. Nicholson-Roberts, A. Phillips, J. Praught, P.S. Rees, I. Schoonbaert, T. Trinick, D.R. Wilson, A.J. Simpson, D. Wang, M.K. O'Shea, T.E. Fletcher

**Affiliations:** a U.K. Defence Medical Services EVD Group, Royal Centre for Defence Medicine, Birmingham, United Kingdom; b Royal Canadian Medical Services, Ottawa, Canada; c Rare and Imported Pathogens Laboratory, Public Health England, Porton, United Kingdom; d Liverpool School of Tropical Medicine, Pembroke Place, Liverpool, L3 5QA, United Kingdom

**Keywords:** Ebola virus disease, Viral haemorrhagic fever, Critical care, Early warning score

## Abstract

**Background:**

Limited data exist describing supportive care management, laboratory abnormalities and outcomes in patients with Ebola virus disease (EVD) in West Africa. We report data which constitute the first description of the provision of enhanced EVD case management protocols in a West African setting.

**Methods:**

Demographic, clinical and laboratory data were collected by retrospective review of clinical and laboratory records of patients with confirmed EVD admitted between 5 November 2014 and 30 June 2015.

**Results:**

A total of 44 EVD patients were admitted (median age 37 years (range 17–63), 32/44 healthcare workers), and excluding those evacuated, the case fatality rate was 49% (95% CI 33%–65%). No pregnant women were admitted. At admission 9/44 had stage 1 disease (fever and constitutional symptoms only), 12/44 had stage 2 disease (presence of diarrhoea and/or vomiting) and 23/44 had stage 3 disease (presence of diarrhoea and/or vomiting with organ failure), with case fatality rates of 11% (95% CI 1%–58%), 27% (95% CI 6%–61%), and 70% (95% CI 47%–87%) respectively (p = 0.009). Haemorrhage occurred in 17/41 (41%) patients. The majority (21/40) of patients had hypokalaemia with hyperkalaemia occurring in 12/40 patients. Acute kidney injury (AKI) occurred in 20/40 patients, with 14/20 (70%, 95% CI 46%–88%) dying, compared to 5/20 (25%, 95% CI 9%–49%) dying who did not have AKI (p = 0.01). Ebola virus (EBOV) PCR cycle threshold value at baseline was mean 20.3 (SD 4.3) in fatal cases and 24.8 (SD 5.5) in survivors (p = 0.007). Mean national early warning score (NEWS) at admission was 5.5 (SD 4.4) in fatal cases and 3.0 (SD 1.9) in survivors (p = 0.02). Central venous catheters were placed in 37/41 patients and intravenous fluid administered to 40/41 patients (median duration of 5 days). Faecal management systems were inserted in 21/41 patients, urinary catheters placed in 27/41 and blood component therapy administered to 20/41 patients.

**Conclusions:**

EVD is commonly associated life-threatening electrolyte imbalance and organ dysfunction. We believe that the enhanced levels of protocolized care, scale and range of medical interventions we report, offer a blueprint for the future management of EVD in resource-limited settings.

## Highlights

• EVD is associated with life-threatening electrolyte imbalance and organ dysfunction.
• Clinical staging/early warning scores can be useful EVD prognostic indicators.
• Enhanced protocolized care is a blueprint for future treatment in low-resource settings.

## Introduction

The recent outbreak of Ebola virus disease (EVD) in West Africa was unprecedented. Following the first case in Guinea in December 2013, 28 616 cases were reported in Guinea, Liberia and Sierra Leone resulting in 11 310 deaths.^1^, ^2^ Fragile healthcare systems in the affected countries struggled to cope with the scale and complexity of the outbreak, and the World Health Organisation declared a Public Health Emergency of International Concern on 8 August 2014, resulting in the mobilisation of the international community to assist in the control of the EVD outbreak in West Africa.^3^

Case finding and contact tracing, combined with safe and dignified burial of corpses, are key public health measures required to control EVD outbreaks, underpinned by social mobilisation.^4^ Case management and isolation of cases in EVD treatment centres is the fourth key pillar of outbreak response.^5^, ^6^ Whilst there were a number of investigational therapies in development and trialled for the treatment of EVD, none were routinely available in West Africa during the recent outbreak. Optimising clinical outcomes therefore was entirely dependent upon the provision of high quality supportive care.

The practical difficulties of providing parenteral fluid and electrolyte therapy in EVD treatment centres, combined with the perceived risks to healthcare workers (HCWs) of invasive clinical procedures resulted in generally poor access to enhanced levels of care across West Africa.^7^, ^8^ During the outbreak in Gulu, Uganda in 2000, higher levels of care were delivered including routine use of intravenous fluids with an overall case fatality rate of 53% (224/425, 95% CI 48%–58%). At the beginning of this outbreak some clinicians also endeavoured to challenge the historically poor EVD patient outcomes with improved supportive care delivery, including the first ETC in Conakry that sustained at a low CFR of 30%–40%.^8^, ^9^ Unfortunately, despite this progress some organisations have failed to learn these key clinical lessons, and still question the benefit and evidence base of intravenous fluid and electrolyte replacement in EVD.^10^

As part of the international response to the EVD outbreak in West Africa, the UK built seven treatment centres in Sierra Leone,^11^ the first of which was commissioned on 5 November 2014 at Kerry Town, near the capital Freetown. This included 20 beds operated by the UK and Canadian Defence Medical Services specifically for HCWs with suspected or confirmed EVD. The EVD treatment unit (EVDTU) was well resourced with dedicated laboratory facilities and was capable of providing high quality medical and nursing care, fluid and electrolyte therapy, blood component transfusion, oxygen therapy and vasopressor support.

Limited data exist describing supportive care management, laboratory abnormalities and outcomes in patients with EVD in West Africa, apart from within EVD clinical trials. Patient age, baseline viral load and renal dysfunction have been highlighted as key prognostic indicators in EVD.^12^, ^13^ Combined analysis of 27 exported cases of EVD to Europe and the United States of America (USA) also highlighted the prevalence of organ dysfunction in EVD and demonstrated the feasibility and importance of critical care interventions, with an associated low case fatality rate of 18.5% (95% CI 6.3%–38%).^14^ We report cohort data from Sierra Leone which is the first description of the provision of enhanced EVD case management protocols in a West African setting, in a unique treatment centre dedicated to caring for infected HCWs. It demonstrates what can be achieved by well-resourced and committed clinical teams and pushes the boundaries of EVD supportive care levels in low-resource settings.

## Methods

### Study design

Demographic, clinical and laboratory data were collected by retrospective review of clinical notes, nursing charts and laboratory records of patients with confirmed EVD admitted to the military EVDTU at Kerry Town, between 5 November 2014 and 30 June 2015. Clinical features, observations and laboratory test results were recorded each day on a standardised proforma. All clinical notes were electronically scanned on patient discharge or death, prior to destruction of the paper copies used in the EVDTU clinical area. All clinical samples and data were collected during routine patient care as part of the public health response and the Sierra Leone ethics and scientific review board confirmed board approval was not required. All information on individual patients was anonymized and recorded on standardised forms, which were kept securely.

### Patients

Patients admitted to the military EVDTU included international and national HCWs and other personnel engaged in the EVD relief effort in Sierra Leone (including doctors, nurses, community healthcare officers, ambulance drivers and hygienists). Local nationals not involved in healthcare were also referred and admitted on a case-by-case basis, although pregnant women and children with EVD were directed to alternative treatment centres. Patients were admitted directly from the community, or were transferred from other treatment centres once their status as a HCW was realised. Diagnosis of EVD was confirmed on admission by EBOV real-time reverse transcriptase polymerase chain reaction (RT-PCR) assay of blood. Baseline data from patients subsequently aeromedically evacuated to Europe or USA is reported, but not included in longitudinal data reported or outcome dependent regression analysis.

### Blood sampling

All patients underwent EBOV RT-PCR testing on admission, provided by the onsite Public Health England laboratory utilising an existing Trombley Ebola Zaire nucleoprotein assay or the Altona RealStar Filovirus RT-PCR Kit (Altona, Hamburg, Germany).^15^ Results were reported as positive or negative, with cycle threshold (Ct) values only being obtained retrospectively. The onsite military laboratory provided basic haematology, clotting, clinical chemistry and blood culture capabilities. On admission, and when clinically indicated thereafter, blood samples were assayed in the onsite laboratory using the Piccolo Express system (Abaxis, CA, USA), Horiba ABX Micros ES60 analyser (Horiba, Montpellier, France) and Hemochron Signature Elite (Accriva Diagnostics,CA, USA). When laboratory facilities were not available, bedside i-STAT® (Abbott Point of Care) testing was undertaken when indicated. The Bact-Alert™ system was used for blood cultures, and if positive a Blood Culture Identification Detection panel was run on the BioFire FilmArray™. A rapid diagnostic test (RDT) was undertaken on admission to exclude malaria (BinaxNOW®). HIV (Alere Determine HIV 1/2 Ag/Ab) and Dengue (SD Bioline Dengue NS1 Ag Ab Combo) rapid diagnostic tests were also available.

### EVD treatment bundle

An EVD treatment bundle was developed to facilitate a protocolized approach to the provision of clinical care thus ensuring optimal utilisation of clinical contact time. Whilst acknowledging there is little evidence of efficacy of any specific intervention in the management of EVD, the elements of the EVD treatment bundle were developed by the clinical group encompassing interventions well established in the management of the critically ill and incorporating basic tenets of care provided in EVD treatment centres. The EVD treatment bundle evolved through regular review by clinical groups at the end of each 60-day roulement.

In the EVD treatment bundle (Annex 1), clinical disease was defined by three stages of illness (Table 1). The main tenets of the EVD treatment bundle include parenteral fluid & electrolyte replacement therapy, stress-ulcer prophylaxis, empirical antibiotics and anti-helminthic medication, analgesia and standardised approaches to the management of coagulopathy & haemorrhage, encephalopathy and shock (Appendix S1). Venous access was routinely achieved by placement of a central venous cannula (CVC) in all patients with stage 2 or 3 disease. Vital signs were recorded 6 hourly with continuous monitoring of heart rate, non-invasive blood pressure and pulse oximetry undertaken when required (and facilitated remotely by closed circuit television monitoring). Urethral catheterisation and placement of faecal management systems were undertaken as clinically indicated. All staff received education in the delivery of the EVD treatment bundle prior to the opening of the EVDTU and prior to each roulement of clinical staff throughout the outbreak.^16^

**Table 1:** Ebola virus disease stage description.

Stage 1	Non-specific febrile illness
Stage 2	Diarrhoea and/or vomiting without organ dysfunction
Stage 3	Diarrhoea and /or vomiting with organ dysfunction: - Acute kidney injury (serum creatinine > ×2 baseline or <0.5 mL/kg /h urine output for 12 consecutive hours: RIFLE classification) - Coagulopathy and/or haemorrhage (abnormal bleeding, or APTT/ACT > ×1.5 upper limit of normal, or PT > ×1.5 upper limit of normal, or platelets <100 × 10/L) - Encephalopathy +/- seizures (any alteration in mental status)

APTT – Activated Partial Thromboplastin Time, ACT – Activated Clotting Time, PT – Prothrombin Time.

Maximum daily early warning scores (national early warning score – NEWS, quick Sepsis Related Organ Failure Assessment – qSOFA) were calculated retrospectively. The qSOFA score can be calculated using basic parameters of clinical assessment and aims to identify patients at increased risk of death due to sepsis.^17^ NEWS is a scoring system (score 0–20) based upon routine clinical observations reflecting the individual's physiological response to illness. When a patient is admitted to a medical facility it can be used to track changes in clinical observations thus alerting the healthcare team to any deterioration and so triggering a timely escalation in clinical care.^18^

### Statistical analysis

Descriptive analyses are reported as frequencies and proportions for categorical variables, means or medians as appropriate for continuous variables. We used Fisher's exact test for comparing categorical variables, and Student's t-test and Mann–Whitney U test for comparing continuous variables. We assessed risk factors for mortality by univariate logistic regression and reported odds ratio of death with its 95% CI. No imputation for missing data was made due to small sample sizes. Hypothesis tests were two-tailed (p < 0.05) and we analysed data with SPSS (version 24).

## Results

### Demographics

During the study period a total of 44 cases were admitted with confirmed EVD. Three international HCWs with confirmed EVD were initially managed in the EVDTU before being evacuated to Europe or the USA for further treatment, all of whom survived. The remaining cases were Sierra Leonean nationals of whom 71% (29/41) were employed in healthcare. The majority of patients were male (28/44) with a median age of 37 years (range 17–63). The mean time from onset of illness until admission was 5.3 days (SD 3.2), with 23/44 patients admitted directly to the EVDTU (Table 2). Excluding those who were aeromedically evacuated, the case fatality rate for those receiving care in the military EVDTU was 49%.

**Table 2:** Demographic characteristics of EVD cases.

	Total (n = 44^a^)	Survived (n = 21)	Died (n = 20)	p-value
Gender				
Male	28 (64%)	11 (53%)	16 (75%)	0.052
Female	16 (36%)	10 (47%)	4 (25%)	
Age (years) Mean (SD)	37.6 (10.9)	35.0 (9.9)	41 (11.7)	0.084
Health care workers	32 (73%)	17 (83%)	12 (60%)	0.18
Non-health care workers	12 (27%)	4 (17%)	8 (40%)	
Nationality				
Sierra Leone nationals	41 (93%)			
Other nationalities	3 (7%)			
Mode of admission				
Direct	23 (52%)	12 (57%)	9 (45%)	0.76
Transfer another ETC	21 (48%)	9 (43%)	11 (55%)	
Time from symptom onset to admission (days) Mean (SD)	5.3 (3.2)	4.71 (3)	6.5 (3)	0.064
Length of admission (days) n = 41^a^ Mean (SD)	7.4 (4.63)	10.2 (4.4)	4.4 (2.7)	<0.001
Time from symptom onset until death/discharge (days) n = 41^a^ Median (range)	13 (5–25)	14 (8–25)	11 (5–17)	0.004

a 44 patients with confirmed EVD were managed at the EVD treatment unit, with 41 admitted until discharge/death (with longitudinal data available). 3 patients aeromedically evacuated to the USA/Europe were not included in outcome analysis. (SD – standard deviation).

### Clinical features

At the time of admission to the EVDTU the most common clinical features were diarrhoea and vomiting (33/44), lethargy (31/44), anorexia (29/44), abdominal pain (28/44) and fever (27/44). Only 3/44 patients had signs or symptoms of haemorrhage at admission, with 10/44 patients reporting hiccups of which 8/10 died. Vital signs at admission were within normal limits in the majority of patients admitted, apart from respiratory rate (mean 24.6, SD 7.5), with mean temperature 37.5°C (SD 1.0) at admission. Other signs and symptoms of EVD patients admitted to the military EVDTU are shown in Table 3. Stage 1 disease (fever & constitutional symptoms only) was present in 9/44 patients, stage 2 disease (presence of diarrhoea and/or vomiting without organ failure) in 12/44 and stage 3 disease (presence of diarrhoea and/or vomiting with organ failure) was present in 23/44 patients with case fatality rates of those not evacuated of 14% (95% CI 1%–58%), 27% (95% CI 6%–61%), and 70% (95% CI 47%–87%) respectively (p = 0.009). Cycle threshold value at baseline was available for 42/44 participants with a mean of 22.7 (SD 5.2) overall, and 20.3 (SD 4.3) in fatal cases and 24.8 (SD 5.5) in survivors (p = 0.007) (Table 3).

**Table 3:** Admission clinical features.

Clinical Features	Total (n = 44)^a^	Survived (n = 21)	Died (n = 20)	p-value
Anorexia	29/44	13/21	15/20	0.342
Abdominal pain	28/44	13/21	15/20	0.132
Back pain	9/44	5/21	4/20	1.0
Conjunctival injection	10/44	5/21	5/20	1.0
Diarrhoea & vomiting	33/44	13/21	19/20	0.006
Dyspnoea	7/44	2/21	5/20	0.217
Delirium/encephalopathy	11/44	2/21	9/20	0.012
Fever	27/44	14/21	11/20	0.539
Haemorrhage	3/44	2/21	1/20	0.583
Headache	25/44	13/21	10/20	0.543
Hiccups	10/44	2/21	8/20	0.027
Lethargy/malaise	31/44	12/21	17/20	0.096
Myalgia	25/44	11/21	12/20	0.766
Arthralgia	19/44	10/21	7/20	0.372
Nausea	23/44	9/21	13/20	0.143
Vital signs at admission				
Pulse – mean (SD)	90.6 (16.9)	89.2 (19)	92 (16)	0.58
Systolic BP – mean (SD)	125 (21.3)	118 (19)	134 (21)	0.02
Temperature – median (range)	37.3 (36.1–39.6)	37.2 (36.1–39.6)	37.4 (36.3–39.2)	0.354
Respiratory rate – median (range)	22 (14–44)	23 (17–44)	22 (14–42)	0.217
Saturations – median (range)	97 (83–99)	96 (83–99)	98 (91–99)	0.02
EVD stage at admission				
Stage 1	9/44	6/21	1/20	
Stage 2	12/44	8/21	3/20	
Stage 3	23/44	7/21	16/20	0.009
Highest EVD stage during admission				
Stage 1	1/41	1/21	0/20	
Stage 2	8/41	8/21	0/20	0.004
Stage 3	32/41	12/21	20/20	
NEWS score at admission Mean (SD)	4.1 (3.5)	3 (1.9)	5.5 (4.4)	0.02
qSOFA score at admission Median (range)	1 (0–3)	1 (0–2)	1 (0–3)	0.359
Ct value at admission (n = 42) Median	20.7 (14.3–37.2)	23.4 (16.2–37.2)	19.6 (14.3–32.1)	0.001

a 44 patients with confirmed EVD were managed at the EVD treatment unit, with 41 admitted until discharge/death. 3 patients were aeromedically evacuated to the USA/Europe. (SD – standard deviation, BP – blood pressure, qSOFA – quick Sepsis Related Organ Failure Assessment, Ct – cycle threshold).

**Fig. 1:**
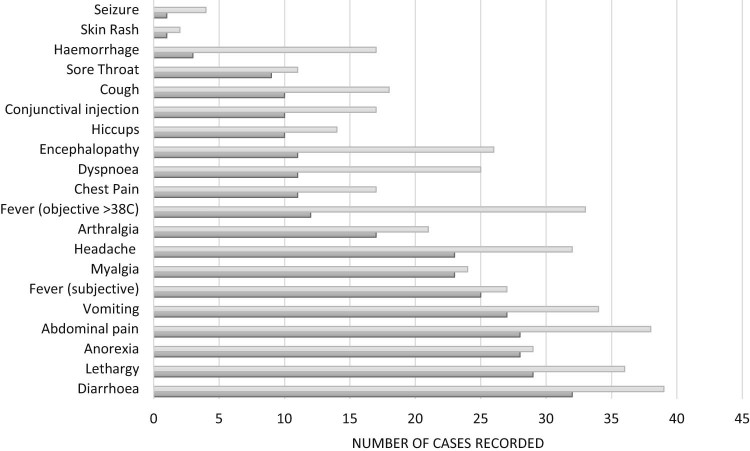
Clinical features during admission (dark grey) and hospitalisation (light grey) (total number = 41).

Clinical findings during hospitalisation are summarised in Fig. 1. Nearly all patients had diarrhoea (39/41), lasting a median 5 days (IQR 3–6 days) and complained of abdominal pain (38/41) lasting a median 3 days (IQR1–3 days). Lethargy was also very common (36/41), lasting a median 3 days (IQR 1–5), with vomiting occurring in 33/41 patients and lasting a median of 3 days (IQR 1–2). During hospitalisation fever (>38 °C) was recorded in 33/41 patients with the median time until resolution (<38 °C) of 9 days from the onset of symptoms. Haemorrhage occurred in 17/41 patients (and one HCW prior to evacuation) with encephalopathy seen in 26/41 patients. The lowest oxygen saturations recorded in fatal cases was lower than cases that survived (mean 86.6% versus 93.9%, respectively, p = 0.003).

### Laboratory results

Blood was analysed using the onsite laboratory and point of care devices as clinically indicated and in accordance with the EVD treatment bundle (e.g. daily in stage 2/3 disease). During a total of 312 EVD patient admission days in the EVDTU, the most frequently measured electrolytes were sodium and potassium, analysed in 255/312 (81.7%) and 240/312 (76.9%) patient days respectively. The majority of patients had electrolyte abnormalities at admission and during hospitalisation (Supplementary Table S1). Hyponatraemia (<135 mmol/L) occurred in 37/40 (93%) of patients and hypokalaemia (<3.5 mmol/L) in 21/40 (53%) during hospitalisation. Hyperkalaemia (>5 mmol/L) occurred in 12/40 patients during admission with 3 patients recording potassium levels >6 mmol/L. Hypomagnesaemia (<0.7 mmol/L) and hypophosphataemia (<0.8 mmol/L) were recognised complications in 11/31 and 8/31 patients in whom magnesium and phosphate were measured, typically occurring in patients with a protracted diarrhoeal phase of illness. Hypoglycaemia (<4 mmol/L) occurred in 24/40 (60%) patients, with 9 patients recording blood sugars ≤2.8 mmol/L, all of whom were in the terminal stages of illness.

Renal dysfunction frequently occurred with an elevated creatinine (>110 umol/L) in 26/41 (63%) at admission and 32/40 (80%) during hospitalisation. Acute kidney injury (AKI) (RIFLE criteria - creatinine > twice estimated baseline) occurred in 20/40 patients through the course of their illness, with 14/20 (70%, 95% CI 46%–88%) subsequently dying, compared to 5/20 (25%, 95% CI 9%–49%) patients who did not suffer AKI dying (p = 0.01). Elevated creatine kinase (>380 IU/L) developed in 33/34 (97%), with the median maximum creatine kinase (CK) observed in patients with AKI 3200 IU/L and in those without AKI, 3129 IU/L. Elevated alanine aminotransferase (ALT) levels (>110 IU/L) were demonstrated in all patients in whom it was measured (35/35), with aspartate aminotransferase (AST) enzyme levels raised in virtually all patients (34/35) during the course of their illness. Peak AST levels (median 1322 IU/L) were higher than peak ALT levels (median 352 U/L). Elevated bilirubin (>21 umol/L) developed in 10/34 patients with 2/34 patients having levels (>100 umol/L). The median C-reactive protein on admission was 30 (n = 34), with 18/35 recording a CRP >200 (limit of assay) during the course of their admission.

Elevated activated partial thromboplastin time (31/35, APPT >37 s) was more common than elevations in prothrombin time (18/39, PT >14 s). Overall, 18/41 (44%) patients developed a significant derangement in one or more assays of global coagulation (Activated Partial Thromboplastin Time (APTT) > 57 s, Activated Clotting Time (ACT) > 180 s or Prothrombin Time (PT) > 23 s) during the course of their illness. Thrombocytopenia (<150 × 10^9^/L) was recorded in the majority of patients (24/35), with significant thrombocytopenia (platelets < 50 × 10^9^/L) observed in 6/35 (17%) patients. The majority of patients with platelets < 50 × 10^9^/L survived (5/6) – only three of whom required platelet transfusion. Leucocytosis (>15 × 10^9^/L) occurred in 16/34 patients and was associated with fatal outcome (p = 0.005).

Malaria RDTs were performed on all patients at admission, with results available in 37 patients, of which one was positive. One patient was also managed empirically for falciparum malaria as a convalescent blood transfusion tested positive by RDT at the time of administration. Thirty-five blood cultures were taken from 27/44 patients, with one positive blood culture identified. Patients were on pre-existing empirical antibiotics in 19/34 negative blood cultures. One positive blood culture (*E. coli* species) was identified from an admission sample of a 48-year-old Sierra Leonean HCW, admitted 7 days after disease onset with severe EVD (stage 3 disease, NEWS 10, qSOFA 2, Ct 18.3). The patient presented with diarrhoea, haemorrhage and acute renal failure and died after 3 days despite supportive therapy including empirical ceftriaxone.

### Early warning scores

At the time of admission mean NEWS score was 5.5 (SD 4.4) in fatal cases and 3 (SD 1.9) in survivors (p = 0.02), with mean qSOFA scores of 1 (SD 0.8) and 0.7 (0.6) respectively (p = 0.27). The highest NEWS score recorded during the course of admission was mean 7.2 (SD 3.7) in survivors vs. 12.8 (SD 3.3) in fatal cases (p < 0.001), with mean highest qSOFA scores also lower in survivors (1.2, SD 0.6) compared to fatal cases (2, SD 0.76, p < 0.001).

### Clinical management

Intravenous catheters were inserted in all patients, with central venous catheters placed in 37/41 patients, and 1/3 international HCWs prior to evacuation (Table 4). The majority CVCs were placed within 24 hours of admission, remaining in situ for a median of 5 days. Faecal management systems were inserted in 21/41 patients during their admission, for a median of 3 days duration (range 1–10 days). Urinary catheters were placed in 27/41 patients for a median duration of 2 days. Intravenous fluid was administered to 40/41 patients for a median duration of 5 days (range 2–17 days). The median maximum IV fluid volume administered to a patient in 24 hours was 3.2 L (range 2-8 L). Intravenous potassium replacement was administered to 31/41 patients, with intravenous magnesium and phosphate replacement administered to 18/41 and 6/41 patients respectively. Blood component therapy was administered to 20/41 patients, and 1 international HCW prior to aeromedical evacuation, with fresh frozen plasma the most frequently administered product. Convalescent whole blood transfusion was administered to 4/41 patients. Empirical ceftriaxone was administered to 37/41 patients for a median of 5 days duration (range 1–10). Oxygen supplementation was administered to 18/41 patients, for a median duration of 3 days (range 1–7), with 16/18 patients having a fatal outcome. Oxygen supplementation was administered to 2 survivors for 5 and 7 days, with one recording a lowest saturation of 77% on room air. It was provided via oxygen concentrators and as such limited to low flow rates. No patients received vasopressors for the management of septic shock. Univariate logistic regression analysis of factors associated with mortality at admission is shown in Table 5. The following laboratory variables were found to be associated with mortality at univariate analysis: AST greater than 1000 U/L (OR 6.0 95% CI 1.17–30.72); RIFLE criteria stage 2 (injury) (OR 4.8 95% CI 1.2–19.13); Granulocyte count greater than 7.5 × 10^9^/L (OR 5.6 95% CI 1.16–27.08); and Ct value less than 20 (OR 19.5 95% CI 3.38–112.05). EVD stage greater than 2 (OR 8.0 95% CI 1.93–33.18) and NEWS greater than 5 (OR 5.19 95% CI 1.28–21.08) at admission were associated with increased mortality.

**Table 4:** Interventions during hospitalisation.

Intervention	Number
Intravenous access	
Peripheral catheter	39 (95%)
Duration (days), median (range) n = 39	4 (1–13)
Central venous catheter	37 (90%)(plus 1/3 exported cases)
Subclavian vein	24 (59%)
Interval jugular vein	11 (27%)
Axillary	1 (2%)
Unspecified	2 (5%)
Central venous catheter inserted within 24 h	31 (76%)
Duration (days), median (range) n = 41	5 (1–10)
Faecal collector system (rectal tube)	21 (51%)
Duration (days), median (range)	3 (1–10)
Urinary catheter	27 (53%)
Duration (days), median (range)	2 (1–10)
Intravenous (IV) fluid	40 (98%)
IV Fluid maximum volume (L) in 24 h (median, range)	3.2 (2–8)
IV Fluid median duration (days, range)	5 (2–17)
IV Fluid median duration in survivors (days, range)	5 (2–17)
Intravenous potassium (K^+^) replacement	31 (76%)
Total K^+^ (mmol) during admission (median, range) n = 31	200 (20–640)
Intravenous magnesium (Mg^2+^) replacement	18 (44%)
Total Mg^2+^ (mmol) during admission (median, range) n = 18	20 (16–156)
Intravenous phosphate (PO_4_^3-^) replacement	6 (15%)
Total PO_4_^3-^ (mmol) during admission (median, range) n = 6	50 (50–250)
Blood component therapy administered	20 (49%)
Fresh frozen plasma	18 (44%)
Cryoprecipitate	9 (22%)
Platelets	8 (20%)
Packed red blood cells	2 (5%)
Intravenous Ceftriaxone	37 (90%)
Median duration, days (range)	5 (1–10)
Oxygen supplementation	18 (44%)
Median duration, days (range)	3 (1–7)

**Table 5:** Factors associated with mortality at admission – univariate logistic regression analysis.

Variable	Odds ratio	95%CI	p-value
Age (year)	0.95	0.89–1.01	0.316
EVD stage >2	8.00	1.93–33.18	0.004
Duration illness pre-admission	0.81	0.64–1.02	0.075
AST >1000 U/L	6.00	1.17–30.72	0.032
RIFLE stage > 2 (injury)	4.80	1.20–19.13	0.002
Granulocyte > 7.5 × 10^9^/L	5.60	1.16–27.08	0.03
Ct value < 20	19.50	3.38–112.05	0.001
NEWS > 5	5.19	1.28–21.08	0.021

AST – aspartate aminotransferase, RIFLE classification (Risk, Injury, Failure, Loss of kidney function, and End-stage kidney), Ct – cycle threshold, NEWS – national early warning score.

## Discussion

The scale of the EVD outbreak in West Africa presented a unique opportunity to improve patient outcomes, our understanding of the natural history of disease and to evaluate novel therapeutics. Much has been learnt from both African cohorts and exported cases in terms of the clinical syndrome of EVD and the benefits of supportive care provision.^19^ The EVDTU at Kerry Town was a small unit dedicated to providing care for HCWs in a well-resourced clinical environment, with high quality nursing care and senior clinician leadership, supported by a comprehensive deployed laboratory. It was committed to improving equity of access to life saving interventions and to pushing the boundaries of EVD supportive care – including central venous catheterisation, ultrasound guided fluid resuscitation, and laboratory support to guide electrolyte replacement.

The majority of patients admitted were HCWs and represent an older and more unwell cohort, compared to other data sets reported. Nearly half of all patients were also transferred from other treatment centres and as such presenting later in their disease course. At admission the majority of the cohort had severe EVD, based on a novel EVD staging system developed by the UK DMS, underpinned by laboratory and clinical data. Although the total number of patients admitted with confirmed EVD was smaller than expected, the unique breadth, depth and completeness of data collected presents a unique opportunity for detailed analysis. It is the first description of an “EVD care bundle” based on established tenets of critical care medicine, and demonstrates the utility of both a novel EVD staging system and established early warning scores in EVD for the first time.

The majority of the patients had renal impairment with 40% having acute kidney injury by RIFLE criteria, 17/40 with serum creatinine >350 umol/L, higher than in other published cohorts.^20^, ^21^ Electrolyte imbalance was extremely common, with hypokalaemia and hypoglycaemia occurring in the majority of patients, and is to be expected with the significant gastrointestinal involvement in EVD. Potassium supplementation was required by a number of patients into the recovery/convalescent phase of illness, and we believe hypokalaemia induced cardiac arrhythmia may be the cause of sudden death in recovering patients that was reported by other clinicians (Dr. M Jalloh – personal communication 2014). Haemorrhage and significant coagulopathy occurred in over 40% of patients, although haemorrhage was not uniformly fatal with 7/17 (41%, 95% CI 18%–67%) patients surviving to discharge. This rate is consistent with aggregate data of exported cases and we believe that it reflects higher staff-to-patient ratios allowing improved case observation, combined with an interventional approach of placing IV catheters. Encephalopathy was also frequently seen, and although it was associated with mortality, 7/26 (27%, 95% CI 12%–48%) patients with encephalopathy survived. The encephalopathy that we observed was an alteration in conscious level accompanied by transient cognitive impairment, consistent with diffuse cerebral dysfunction that was hypoactive in nature. We believe the aetiology is most likely multifactorial, incorporating metabolic and infective factors with direct viral brain involvement possibly contributing.^22^ Identifying features which reliably predict poor clinical outcome at presentation and during the course of illness improve understanding of disease pathogenesis and can inform future intervention strategies. In our cohort, factors significantly associated with poor outcome at presentation include a low Ct value, presence of established organ dysfunction (EVD stage 3) and a high NEWS score. Other significant laboratory variables associated with mortality in univariate analysis at baseline include AST, ALT, creatinine, CRP and white blood count. Recent further analysis of clinical trial data also showed significant electrolyte abnormalities in their EVD cohort and tested a prognostic model also incorporating calcium and haemoglobin levels.^23^

Early warning scores aim to identify patients at risk of severe illness or death, utilising simple physiological parameters and have long been advocated by critical care outreach and acute medical teams.^18^ In keeping with other forms of critical illness, timely intervention with supportive care before organ dysfunction becomes established and has the potential to improve clinical outcomes in EVD. Due to the limited provision of systematic vital signs monitoring and data recording in EVD patients in West Africa, limited data existed to inform development of EVD early warning systems. We retrospectively analysed our data utilising the UK national early warning score system and qSOFA scores at baseline, that in comparison to the novel staging system we report they do not rely on laboratory parameters. Admission NEWS score was significantly higher in fatal cases, and could be utilised in the future as a prognostic indictor and planning tool. The qSOFA showed no utility in predicting outcome in EVD in our cohort. Although our data set is small it provides the first evidence of the utility of an early warning score in EVD and requires evaluation in larger cohorts, ideally prospectively.

The scale and range of medical interventions we report highlights what is feasible in deployed low-resource settings. Central venous access requires skill, but facilitates fluid and concentrated electrolyte replacement, limiting invasive procedures.^24^ Peripherally inserted central lines may provide an alternative, dependent on operator skill and experience and were used successfully in 40% of exported cases.^14^ There were no needlestick injuries associated with central venous catheters in our staff, and one healthcare worker infection in an EVDTU nurse, the cause of which was not identified. Faecal management systems were also used for the first time in patients with EVD in West Africa. They were well tolerated by patients and used in 50% of cases, providing infection prevention and control benefits for staff and cleanliness and dignity for patients with profuse diarrhoea. In this cohort of patients with EVD, hypotension refractory to fluid resuscitation in keeping with the clinical syndrome of septic shock was not observed and as such vasopressors were not utilised. We were also fortunate to have access to comprehensive panel of blood products that were delivered to almost 50% of patients. The EVDTU was not a clinical trial site for novel therapeutics during the outbreak and focussed on delivery of high quality supportive care. However, at their own request, and through provision by referring units, three HCWs received convalescent whole blood transfusion, providing informed consent and ultimately all surviving their infection. Subsequent clinical trial data failed to show any clear benefit of convalescent plasma in EVD.^25^ Experimental antiviral therapies had not been used for post-exposure prophylaxis (PEP) in any of this cohort, but a number of international HCWs did receive (PEP) after high risk exposures and evacuation to the UK.^26^

Whilst we rightly emphasise the benefits of comprehensive supportive care for EVD patients we must also recognise the benefits of palliative care and psychological support in patients who will not survive. We had established protocols for end of life care and with higher staffing levels, were able to provide improved symptom management to fatal cases with 75% receiving opiate analgesia and 50% benzodiazepines, frequently through syringe drivers. Opiate analgesia was also utilised in over 50% of survivors, mainly as an oral formulation.

Healthcare workers at the forefront of the fight against EVD have suffered consistently high case fatality rates. The reasons underlying these rates across the outbreak remain unclear, but older age, comorbidities and high viral load are consistently associated with poorer outcome and may be more common in HCWs. A clinical prioritization score developed by Hartley et al.,^27^ adjusted for age and viral load predicted a case fatality rate of 62% in our cohort (95% CI 45%–74%) at admission. The case fatality rate in the military EVDTU was 49% (95% CI 33%–65%) overall, but direct comparison of clinical outcomes from different treatment facilities has proved difficult. Rates reported from other ETCs in Sierra Leone were similar from cohorts with lower median ages and higher Ct values. International Medical Corps^28^ reported a case fatality rate of 58% (95% CI 53%–64%, median Ct 25.4), and Medicins san Frontieres,^29^ a case fatality rate of 51% (95% CI 47%–56%, median Ct 31 in survivors and 22 in fatal cases). Historical control data for 3 months preceding the JIKI trial in Guinea had similar Ct values (Adults and children ≥6 years Ct value 20.1) with a case fatality rate of 57% (272/478, 95% CI 52%–61%).^30^ A study of the heterogeneity in the case fatality rates in West Africa,^31^ was limited by missing outcome data, but demonstrated that in the age group 35–39 years there was a case fatality rate of 67.5% (95% CI 62%–72%, n = 351). Analysis of supplementary data from this study also showed that in adults (≥17 years, median age 32 years) with confirmed EVD and known outcome status admitted to ETCs in Sierra Leone, there were 268/477 deaths (CFR 56%, 95% CI 52%–61%). Whilst the difficulty of comparing outcomes in cohorts with significantly different demographics and baseline viral loads is obvious, the importance of survivor bias due to local factors affecting patient distribution must also be considered.^32^

A number of limitations of our data must be appreciated, that reflect the primary mission of the EVDTU to manage infected HCWs. As such paediatric or pregnant cases were not referred to the EVDTU, that may have different clinical syndromes and management approaches. The EVDTU was also highly resourced with its main assets being staff-to-patient ratios and laboratory support. The intensive medical and nursing care provided would be challenging to safely maintain in larger ETCs, but a compromise incorporating the tenets of our management approach is achievable. The number of HCW cases that were admitted was also lower than expected resulting in a small sample size. This reflects the timing of the epidemic and reduced nosocomial risk to HCWs as better treatment centres opened, and is consistent with the experience of other HCW ETCs in Guinea and Liberia. Small sample size has prevented us from performing multivariable analysis to looking at the risk factors of mortality at admission simultaneously. As a result, observed differences may be subject to possible confounding effects due to unknown or unmeasured factors.

In resource-limited African settings, the provision of even basic supportive care to patients with EVD is difficult. Recommendations to limit treatment based upon perceived poor patient prognosis and risk to HCWs, perpetuate the cycle of limited care, poor outcomes and fear.^33^ Whilst the provision of renal replacement therapy and mechanical ventilation have been successfully utilised in one ETC in Sierra Leone during the recent outbreak, the logistic and practical considerations of providing level 3 care, unfortunately makes it an unrealistic proposition as an established level of care for EVD in future outbreaks. We do recognise that when resources and appropriately trained personnel exist, this capability can be safely delivered and will improve outcomes in severe EVD for limited numbers of patients. We believe that the approach pioneered at our EVDTU utilising improved resources, clinical staging of disease severity and an enhanced level of protocolized care offers a blueprint for the future management of EVD in resource-limited environments. These processes were subsequently adopted by other non-governmental organisations in West Africa, and can form a platform for future viral haemorrhagic fever clinical care augmented by specific therapeutics when available.

## Conflict of interest

The authors declare that they have no competing financial interests. The content is solely the responsibility of the authors.

## Funding

No specific funding. TF is funded by the Wellcome Trust (104480/Z/14/Z) and the UK Ministry of Defence. The PHE-led EVD laboratory operation was funded through the Department for International Development.

## Appendix A Supplementary data

Table S1 Abnormal laboratory values at admission or at any time during hospitalization. Table S1
