# Supplementary material for: Enhanced case management can be delivered for patients with EVD in Africa: Experience from a UK military Ebola treatment centre in Sierra Leone
Source: J Infect. 2018 Apr;76(4):383–92. doi: 10.1016/j.jinf.2017.12.006 (PMC5903873; doi:10.1016/j.jinf.2017.12.006)
Supplement: Table S1 — Abnormal laboratory values at admission or at any time during hospitalization. [file mmc1.docx]

Supplementary Table 1 - Abnormal laboratory values at admission or at any time during hospitalization

| **Abnormal laboratory result** | **At Admission** | | | | **During hospitalisation** | | | |
| --- | --- | --- | --- | --- | --- | --- | --- | --- |
| l | Total | Survived | Died | Test – p-value | Total | Survived | Died | Test – p-value |
| **Hyponatraemia**  (<135 mmol/litre) | 31/43 (71%) | 14/21 (67%) | 15/19 (79%) | 0.49 | 37/40 (93%) | 20/21 (95%) | 17/19 (89%) | 0.6 |
| **Hypokalaemia**  (<3.5 mmol/litre) | 8/43 (19%) | 4/21 (19%) | 3/19 (16%) | 1.0 | 21/40 (53%) | 10/21 (45%) | 11/19 (58%) | 0.55 |
| **Hyperkalaemia**  (>5 mmol/litre) | 5/43 (12%) | 2/21 (9.5%) | 3/19 (16%) | 0.65 | 14/40 (30%) | 6/21 (29%) | 8/19 (42%) | 0.51 |
| **Hypomagnesemia**  (<0.7 mmol/litre) | 3/25 (12%) | 2/14 (14%) | 0/8 (0%) | 0.52 | 11/31 (35%) | 9/20 (45%) | 2/11 (18%) | 0.24 |
| **Hypophosphatemia**  (<0.8 mmol/litre) | 2/25 (8%) | 1/14 (7%) | 1/8 (13%) | 1.0 | 8/31 (26%) | 7/20 (35%) | 1/11 (9%) | 0.2 |
| **Hypoglycaemia**  (<4 mmol/litre) | 7/43 (16%) | 2/21(9.5%) | 5/19 (26%) | 0.23 | 24/40 (60%) | 12/21 (57%) | 12/19 (63%) | 0.76 |
| **Hypoalbuminaemia**  (<35 g/litre) | 26/34 (76%) | 15/19 (79%) | 10/11 (91%) | 0.63 | 34/34 (100%) | 20/20 (100%) | 14/14 (100%) | 1.0 |
| **Elevated creatinine** >110 umol/litre | 26/41 (63%) | 7/19 (37%) | 19/19 (100%) | <0.0001 | 32/40 (80%) | 13/21 (62%) | 19/19 (100%) | 0.004 |
| **Elevated creatinine**  > 350 umol/litre  (RIFLE3 – Acute kidney failure) | 14/41 (34%) | 3/19 (16%) | 11/19 (58%) | 0.02 | 17/40 (43%) | 5/21 (24%) | 12/19 (41%) | 0.02 |
| **Elevated aspartate aminotransferase – AST** (>98 U/litre) | 26/33 (78%) | 14/18 (78%) | 10/11 (91%) | 0.62 | 33/34 (97%) | 20/20 (100%) | 13/14 (93%) | 0.41 |
| **Elevated alanine aminotransferase** – **ALT** (>110 U/litre) | 24/34 (71%) | 12/18 (67%) | 12/13 (92%) | 0.19 | 35/36 (97%) | 20/20 (100%) | 15/16 (94%) | 0.44 |
| **Hyperbilirubinaemia** (>21 umol/litre) | 4/34 (12%) | 0/19 (0%) | 4/12 (33%) | 0.02 | 10/35 (29%) | 2/20 (10%) | 8/15 (53%) | 0.008 |
| **Elevated creatinine** **kinase** (>380IU/L) | 27/33 (82%) | 15/17 (88%) | 12/13 (92%) | 1.0 | 33/34 (97%) | 19/19 (100%) | 14/15 (93%) | 0.44 |
| **Anaemia**  (<11 g/dL) | 1/42 (2%) | 0/20 (0%) | 1/20(5%) | 1.0 | 13/40 (33%) | 7/21 (33%) | 6/19 (32%) | 1.0 |
| **Thrombocytopenia**  Plts <150,000/uL Plts <50,000/uL | 20/34 (59%)  1/34 (29%) | 14/19 (74%)  1/20 (5%) | 3/12 (25%)  0/12 (0%) | 0.01  1.0 | 24/35 (69%)  6/35 (17%) | 18/20 (90%)  5/20 (25%) | 6/15 (40%)  1/15 (7%) | 0.003  0.21 |
| **Leucopenia**  (WBC <3500/uL) | 10/34 (29%) | 6/19 (32%) | 1/12 (8%) | 0.2 | 10/34 (29%) | 9/20 (45%) | 1/14 (7%) | 0.02 |
| **Leucocytosis** (WBC >15,000uL) | 7/34 (21%) | 2/19 (11%) | 5/12 (42%) | 0.08 | 16/34 (47%) | 5/20 (25%) | 11/14 (79%) | 0.005 |
| **Granulocytopenia** (<2500/uL) | 11/34 (32%) | 8/19 (42%) | 1/12 (8%) | 0.1 | 11/35 (31%) | 9/20 (45%) | 2/15 (3%) | 0.07 |
| **Lymphopenia** (<1500/uL) | 13/33 (39%) | 8/19 (42%) | 2/12 (17%) | 0.24 | 16/34 (47%) | 14/20 (70%) | 2/14 (14%) | 0.002 |
| **Elevated Prothrombin time** (>14s) | 9/37 (24%) | 2/18 (11%) | 7/16 (44%) | 0.052 | 18/39 (46%) | 5/21 (24%) | 13/18 (72%) | 0.004 |
| **Elevated activated partial thromboplastin time** (>37s) | 25/33 (76%) | 12/18 (67%) | 12/12 (100%) | 0.057 | 31/35 (89%) | 17/21 (81%) | 14/14 (100%) | 0.13 |
