# Supplementary material for: Enhanced case management can be delivered for patients with EVD in Africa: Experience from a UK military Ebola treatment centre in Sierra Leone
Source: J Infect. 2018 Apr;76(4):383–92. doi: 10.1016/j.jinf.2017.12.006 (PMC5903873; doi:10.1016/j.jinf.2017.12.006)
Supplement: Appendix S1 — Supplementary file. [file mmc2.pdf]

Ebola Virus Disease (EVD)

Clinical Guidelines 3.0

EVD – Suspect case criteria

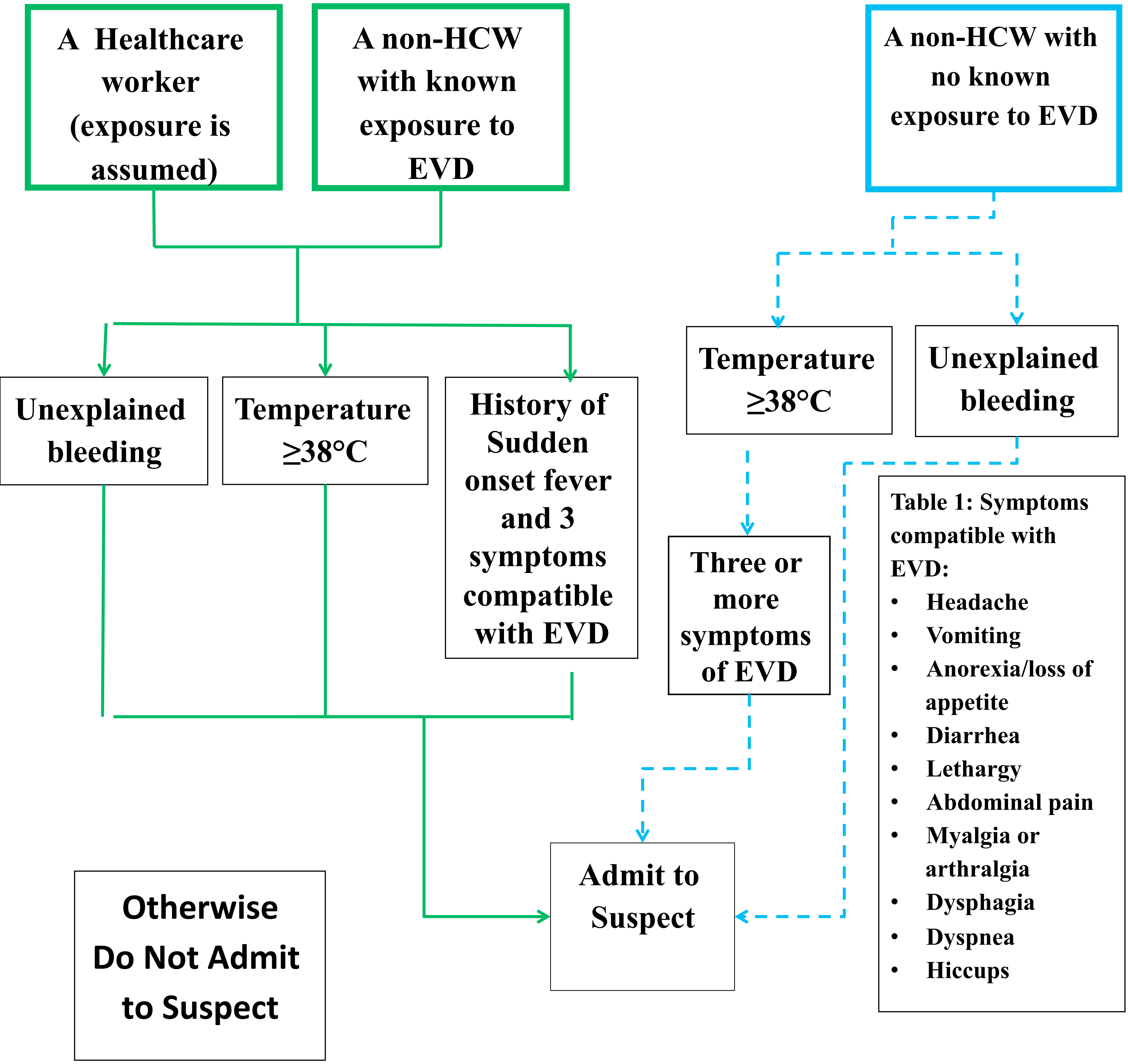

EVD- Stages of Illness

**Stage 1**	Non-specific febrile illness
**Stage 2**	Diarrhoea and/or vomiting without organ dysfunction
**Stage 3**	Diarrhoea and /or vomiting with organ dysfunction:<br>- acute kidney Injury<br>(serum creatinine > X2 baseline or<br><0.5 ml/kg/hr urine output for 12 hours)<br>- coagulopathy and/or haemorrhage<br>(abnormal bleeding, or PT or APTT or ACT > x1.5 upper limit normal)<br>- any alteration in mental status (encephalopathy +/- seizures)<br>- shock (SBP<90 or MAP<70) that is unresponsive to fluid challenges

EVD-TU Admission Bloods

- In all patients:
  - Full blood count, PT & APTT
  - Amylyte 13 - urea & electrolytes, LFTs, calcium, CK, amylase, CRP, glucose
  - Blood cultures
  - Malaria rapid diagnostic test (RDT)
    - if first malaria RDT negative consider repeat test at 24 hours (and consider Dengue RDT)
  - Ebola Virus PCR
    - repeat PCR 72 hours from onset of fever if first test negative
- In selected patients:
  - if diarrhoea and vomiting present
    - Metlac 12 - magnesium, phosphate, lactate, bicarbonate, chloride
  - if shock / haemorrhage present
    - venous blood gas (iSTAT CG4 cartridge)

EVD - Stage 1 Illness

**Clinical Management**<br><br>* Anti worming therapy only given to patients at risk of worms	• oral rehydration solution<br>• paracetamol 1g po 6 hourly / PRN<br>• ivermectin 200mcg/kg po daily for 2 days *<br>• albendazole 400mg po stat *<br>• oral zinc 20 mg po daily<br>• multivitamin tab one po daily
**Observations**	• twice daily observations<br>- temperature<br>- pulse, blood pressure, O_2 saturations<br>- respiratory rate<br>- AVPU<br>• record volume of oral intake
**Investigations**	• admission blood tests<br>• none routine
**Intervention**	• peripheral cannula

EVD Stage 2 Illness

**Clinical Management**<br><br>Physiological targets:<br>- systolic BP >100mmHg<br>- O_2 saturations >92%<br>- urine output >0.5ml/kg/hr<br><br>* Anti worming therapy only given to patients at risk of worms	▪ continue to encourage oral rehydration solution<br>• iv fluid management - Ringers Lactate<br>• iv electrolyte replacement - K, Mg & phosphate<br>• supplemental oxygen therapy if required<br>• paracetamol 1g po/iv 6 hourly /PRN<br>• ivermectin 200mcg/kg po daily for 2 days *<br>• albendazole 400mg po stat *<br>• oral zinc 20 mg po daily<br>• multivitamin one po daily<br>• ranitidine 50mg iv 12 hourly<br>• vitamin K 10mg iv daily<br>• consider metoclopramide 10mg iv 8 hourly and/or ondansetron 4mg iv 8 hourly<br>• consider ceftriaxone 2g iv daily if possible bacterial sepsis
**Observations**	• six-hourly - temperature<br>- pulse,blood pressure, O_2 saturations<br>- respiratory rate<br>- AVPU<br>• record fluid-balance & stool frequency and volume
**Interventions**	• consider: - early central venous cannula<br>- urinary catheter<br>- bowel management system
**Bloods**	• daily - FBC, PT, APTT,<br>- Amylyte 13 / Metlac 12 on alternate days<br>• blood cultures - before commencing antibiotics

EVD Stage 3 Illness

**Clinical Management**<br><br>Physiological targets:<br>- systolic BP >100mmHg<br>- O_2 saturations >92%<br>- urine output >0.5ml/kg/hr<br><br>* Anti worming therapy only given to patients at risk of worms	▪ continue to encourage oral rehydration solution<br>• iv fluid management - Ringers Lactate<br>• iv electrolyte replacement - K, Mg & phosphate<br>• supplemental oxygen therapy if required<br>• paracetamol 1g po/iv 6 hourly / PRN<br>• ivermectin 200mcg/kg po daily for 2 days *<br>• albendazole 400mg po stat *<br>• oral zinc 20 mg po daily<br>• multivitamin one po daily<br>• ranitidine 50mg iv 12 hourly<br>• vitamin K 10mg iv daily<br>• consider metoclopramide 10mg iv 8 hourly and/or ondansetron 4mg iv 8 hourly<br>• consider ceftriaxone 2g iv daily if possible bacterial sepsis<br>• management of coagulopathy & haemorrhage<br>• management of encephalopathy & seizure<br>• management of shock
**Observations**	• six-hourly - temperature<br>- pulse, blood pressure, O_2 saturations<br>- respiratory rate<br>- AVPU<br>• fluid-balance & stool frequency and volume
**Interventions**	• consider: - central venous catheter<br>- urinary catheter<br>- bowel management system
**Bloods**	• daily - FBC, PT, APTT<br>- Amylyte 13 /Metlac 12 on alternate days<br>• take blood cultures at onset of shock

Management of electrolyte abnormalities & hypoglycaemia in EVD

**Hypokalaemia**<br>(Target range 3.5 - 4mmol/L)	• peripheral cannula (if no CVC)<br>- 40 mmol KCL in 1000ml 0.9% saline over > 2 hours<br>• central venous catheter<br>- 40mmol KCl in 100ml 0.9% saline over 2 hours
**Hyperkalaemia**	• if K > 6.0mmol/L on laboratory blood test<br>- check iSTAT to confirm hyperkalaemia<br>- 10ml calcium chloride 10% iv over 5 min<br>- 10 units of actrapid insulin in 300ml 10% Dextrose over 60 min<br>- 1.26% sodium bicarbonate infusion 100ml/h<br>• re-check potassium on iSTAT after 2 hours
**Hypomagnesaemia**	• if Mg < 0.7mmol/L<br>- 5g (20 mmol) magnesium sulphate in 50ml 0.9% saline over 2 hours
**Hypophosphataemia**	• if PO_4 < 0.7 mmol/L<br>- Phosphate (Polyfusor) 50mmol in 500ml over 6 -12 hours
**Hypoglycaemia**	• if blood glucose < 5mmol/L commence 10% Dextrose infusion 30ml/hr - monitor blood glucose and adjust infusion as required<br>• consider hypoadrenalism

Management of EVD-related shock

**Optimisation of intra-vascular volume**	• clinical assessment - consider haemorrhage<br>• observations - pulse, blood pressure, urine output<br>• review fluid balance<br>• dynamic ultrasound assessment of inferior vena cava<br>• venous blood gas (iSTAT CG4) to assess lactate & central venous O_2 saturations<br>• maintain urine output >0.5ml/kg/hr
**Vasopressor**<br>(to be initiated only following appropriate clinical review and subject to safe staffing levels)	• noradrenaline 8mg made up to 50ml in 5% Dextrose via central venous cannula (DO NOT BOLUS).<br>- continuous monitoring of ECG and O_2 saturations<br>- NIBP - every 5 mins when unstable<br>- every 30 mins when stable<br>- aim for Mean Arterial Pressure (MAP) > 65mmHg<br>MAP = DBP + (1/3 x (SBP-DBP))
**Adjunctive steroid therapy**	If noradrenaline dose >0.4 mcg/kg/min then commence Hydrocortisone 50mg iv 6 hourly

Ready reckoner for noradrenaline infusion dosing (mcg/kg/min)

Noradrenaline infusion rate (ml/hour)
(8mg/50ml or 160mcg/ml)

Body Weight (kg)

	1	2	3	4	5	6	7	8	9	10	11	12	13	14
**30**	0.09	0.17	0.26	0.35	0.44	0.53	0.62	0.71	0.80					
**35**	0.08	0.15	0.23	0.30	0.38	0.46	0.53	0.60	0.69	0.76	0.84			
**40**	0.06	0.13	0.20	0.26	0.33	0.40	0.46	0.53	0.60	0.66	0.73	0.80		
**45**	0.06	0.12	0.18	0.24	0.30	0.36	0.41	0.47	0.53	0.59	0.65	0.71	0.77	
**50**	0.05	0.10	0.15	0.21	0.26	0.32	0.37	0.43	0.48	0.53	0.59	0.64	0.69	0.75
**60**	0.04	0.09	0.13	0.18	0.22	0.27	0.31	0.36	0.40	0.44	0.49	0.53	0.58	0.62
**70**	0.04	0.08	0.11	0.15	0.19	0.23	0.27	0.30	0.34	0.38	0.42	0.46	0.50	0.53
**90**	0.03	0.06	0.09	0.12	0.15	0.18	0.21	0.24	0.27	0.30	0.33	0.36	0.39	0.42

add adjunctive steroid therapy

Management of EVD coagulopathy, haemorrhage & VTE prophylaxis

**Haemoglobin**	• in the presence of active bleeding transfuse Packed Red Blood Cells & Fresh Frozen Plasma (1:1 ratio)<br>• target haemoglobin >9g/dl
**Platelets (when available and clinically indicated)**	• transfuse 1 adult therapeutic dose of pooled platelets when indicated<br>• target platelet count:<br>>20 x 10^9 /L in the absence of bleeding<br>>50 x 10^9/L in the presence of bleeding
**PT / APTT**	• in the presence of bleeding transfuse ~15ml/kg Fresh Frozen Plasma 12 hrly<br>• minimise crystalloid infusions as diarrhoea permits<br>• transfuse 1 unit of cryoprecipitate for every 6 units of Fresh Frozen Plasma<br>• continue vitamin K 10mg iv 24 hourly until coagulopathy resolves (stop after 3 days if no coagulopathy)<br>• do not routinely attempt to correct abnormal PT / APTT with Fresh Frozen Plasma in the absence of bleeding
**Hyperfibrinolysis**	• in the presence of persistent bleeding consider<br>- tranexamic acid 1g iv 8 hourly
**Gastrointestinal haemorrhage**	• in the presence of suspected or confirmed gastrointestinal haemorrhage commence omeprazole 80mg iv over 1 hour, then 40mg bolus iv 12 hourly
**VTE prophylaxis**	• In the recovery phase and in the absence of haemorrhage/coagulopathy, commence enoxaparin 40mg s/c daily (20mg if eGFR <30ml/min) until patient is ambulatory

Management of EVD-related encephalopathy & seizures

Consider and treat underlying pain causing agitation.	
Personal safety of the healthcare worker is paramount - only undertake clinical interventions in the confused patient when safe to do so.	
**Mild confusion**	• haloperidol 2.5-5mg iv stat PRN<br>• lorazepam 1-2mg iv stat PRN
**Moderate confusion**	• haloperidol 5mg iv stat4-6 hourly<br>• lorazepam 1-2mg iv stat 6-12hourly
**Severe confusion**	• midazolam infusion :50mg made up to 50ml in 0.9% saline - 2-5ml/hr<br>• haloperidol 2.5-5mg iv 4-6 hourly<br>• consider appropriate physical restraint in addition to sedation in the severely confused patient
**Seizure**	• lorazepam 1mg iv stat - repeat after 5 min if still fitting<br>• phenytoin 20mg/kg in 250ml 0.9% saline (max 2g) over 30 min with ECG monitoring

EVD - Management of Pain Algorithm

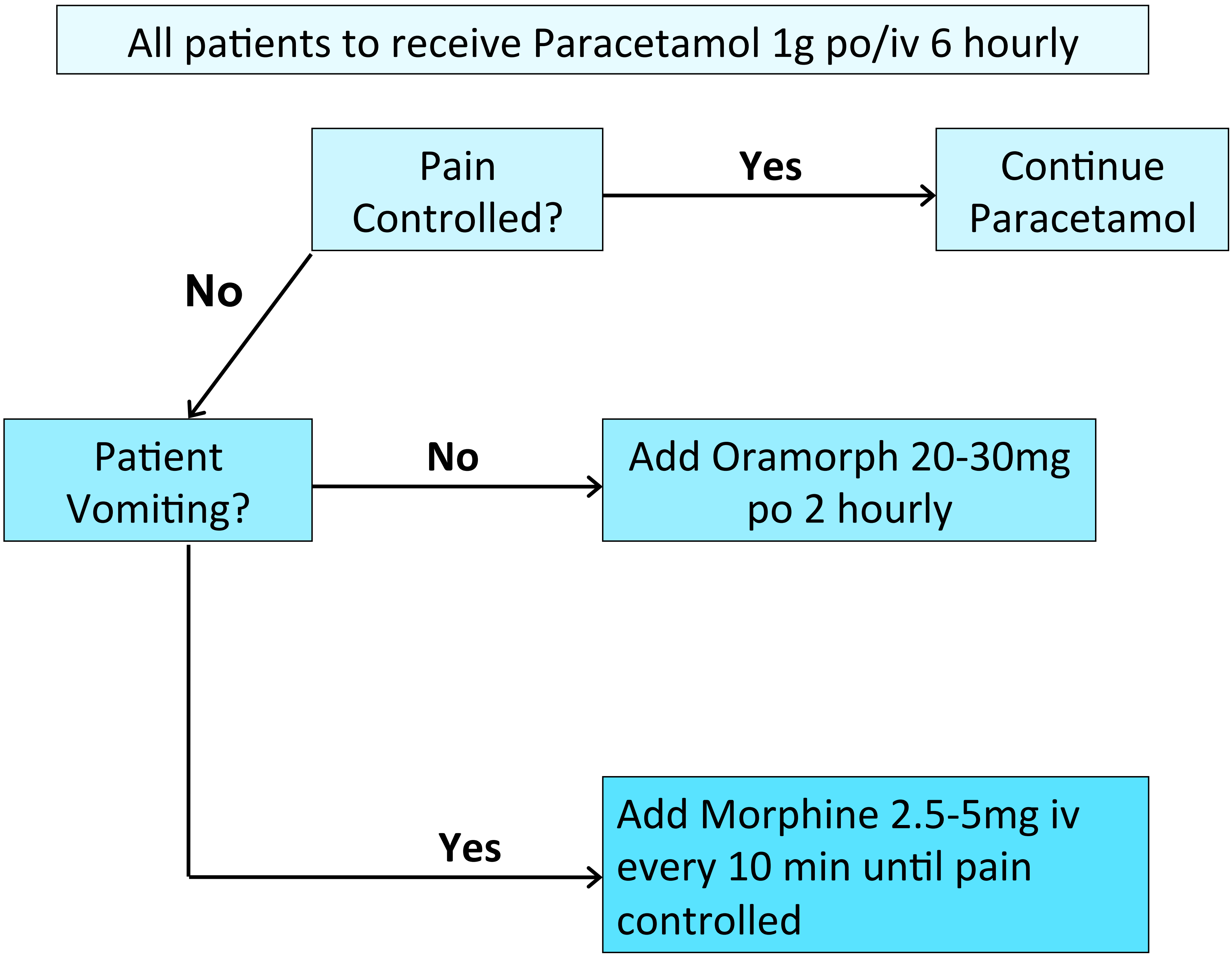

Considerations:

- Naloxone 200-400mcg iv stat should be prescribed PRN in all patients receiving opioids
- Antiemetics should be prescribed in patients receiving opioids
- NSAIDs are not to be used (due to the risk of bleeding and renal failure)
- Alternative or adjuvant analgesia should be considered by medical staff

Patients with end stage disease will be considered on a case by case basis and receive sufficient doses of analgesia and anxiolytics in order to maintain comfort

EVD Nutritional assessment & support

**1. Weigh patient at admission**	Body mass index may also be calculated
**2. Weigh patient on discharge**	For use at survivor clinics and for follow up
**3. Approach to treatment**	Most EVD patients have a high risk of malnutrition with a high total malnutrition risk score of 2 or 2+<br>•Encourage high protein/ calorie snacks<br>•Prescribe Oral Nutritional Supplements (Plumpy'Nut ® 1-2/day OR Ensure 2/day)<br>•Daily Multivitamin
**4. Anticipate & prevent refeeding syndrome# (RFS)**<br><br>#metabolic disturbances relating to the reinstitution of nutrition Can cause cardiac arrhythmias, sudden death, coma, confusion, heart failure	1. Check & <u>actively correct</u> baseline K/PO4/Mg/Ca - only withhold replacement if levels high<br>2. Ensure adequate thiamine and B vitamins: Consider IV Pabrinex- 1 pair of ampoules od or high dose thiamine (300 mg/day) & Vit B CoStrong (1-2 tablets/day).<br>3. Ensure on multivitamin daily<br>4. Actively monitor glucose levels -be cautious with 10% dextrose replacement as ↑ risk of RFS<br>5. Monitor - K, PO4, Ca, Mg on at least alternate days (preferably daily) during first week

End-of-life care in patients with EVD

**On-going medication**	• Drugs that do not contribute to end-of-life treatment goals (such as antibiotics and replacement electrolytes) should be stopped.
**Intravenous fluids and food**	• Aggressive i.v. fluid resuscitation should cease.<br>• Maintenance fluids and food can be stopped if appropriate, however they are unlikely to significantly prolong life in end-stage EVD.<br>• Oral fluids and regular mouth-care should be offered for thirst.
**Invasive lines and procedures**	• The duty medical officer should weigh-up the potential benefits to the patient against the risks to healthcare staff prior to undertaking procedures or removing lines.
**Blood tests**	• Should not be performed.
**Symptom control**	• **Pain**: Morphine infusion 1-2.5mg/hr i.v. is a suitable starting dose in an opioid-naïve patient (50mg made up in 50ml 0.9% saline). Consider an initial bolus and titrate to effect. Paracetamol 1g qds i.v. should be continued.<br>• **Dyspnoea**: morphine (as above). Oxygen therapy (up to 5L/min via O_2 concentrator) may be beneficial where hypoxia is a contributory factor<br>• **Delirium**: Midazolam infusion 1-2mg/hr (50mg made up in 50ml 0.9% saline). Titrate to effect. Also consider dimming lights and reducing noise.<br>• **Nausea**: Ondansetron 4-8mg iv tds prn +/- Metoclopramide 10mg iv tds prn<br>• **Diarrhoea**: Loperamide 4mg iv qds<br>• **Respiratory secretions/bowel colic**: Hyoscine butylbromide 20mg s.c. qds prn
**Next-of-kin**	• Every effort must be made to contact the patient's next-of-kin and keep them informed throughout the process.
**After death**	• Follow procedures as detailed in the 'Care of the Deceased' SOP.

When the medical team decide to withdraw life-prolonging treatment, this decision should be carefully documented in the medical notes and the DMD informed.

Body fluids of patients with EVD who are approaching death will have a high viral load and it is particularly important that exposure-prone procedures are minimised to protect healthcare staff. For this reason intravenous infusions are favoured over bolus medication and subcutaneous infusions are not used.

Convalescent Stage – Recovering from EVD

**Clinical Management**	• Encourage oral fluids and diet<br>• Encourage mobilisation at least once per shift<br>• Encourage use of bathroom instead of commode<br>• Consider Enoxaparin 40mg SC OD until mobilising<br>• Consider Paracetamol 1g PO QDS PRN for pain<br>• Consider Ondansetron 4mg PO TDS PRN for nausea
**Observations**	• Twice Daily Observations:<br>- Blood Pressure - Pain (out of 3)<br>- Pulse - SpO_2<br>- Respirations - AVPU<br>- Temperature<br>• Observe for any areas of skin breakdown and treat accordingly
**Invasive devices**	• Consider removal of CVC / peripheral iv access<br>• Consider removal of urinary catheter and bowel management system
**Blood tests**	• No routine blood tests required unless on iv fluids<br>• EVD PCR: performed once patient asymptomatic for 72 hours. If result is negative, patient should be discharged.

Patients who are asymptomatic for EVD defining symptoms are considered to be convalescing. When denoting EVD stage use the highest stage reached but prefixed with a ‘C’

Discharge Criteria 1 of 2

SURVIVORS

1.Patients with confirmed EVD who have recovered and are medically fit require a safe discharge process to ensure no cross contamination from the high risk area.

Criteria for discharge:

a.Asymptomatic, tolerating oral fluids, afebrile and without haemorrhage for >72hours with a negative PCR for Ebola Virus Disease (EVD).

b.Patients who meet part a, but are not yet fit for discharge into the community. Refer to SOP 032 STEP-DOWN.

c.Patients with significant symptoms, not thought to be due to EVD, require 2 negative PCRs 24hrs apart.

d.Persistent low levels of viraemia (two EVD PCR 24 hours apart each with CT>38) in patients who have been asymptomatic at the time of the first of these EVD PCRs for >72 hours should be discussed with local PHE scientists. It may be possible to discharge them but this needs to be considered on a case-by-case basis.

Discharge Criteria 2 of 2

SUSPECT CASES

1.A suspect case admitted to the EVD TU can be considered negative for EVD when they have a negative EVD PCR at 72hr from onset of symptoms. Normally, any patient who has a negative EVD PCR before 72hr requires a further test at 72hr from onset of symptoms.

2.There may be situations where an alternative diagnosis is confirmed, which negates the need for the 72 hr EBOV PCR.

3.These should all be discussed with the ID specialist on call and the DMD, and the plan disseminated to the CP and the wider clinical team.
